# Pregnancy outcomes and neonatal thyroid function in women with thyroid cancer: a retrospective study

**DOI:** 10.1186/s12884-023-05588-4

**Published:** 2023-05-25

**Authors:** Xianxian Yuan, Jinqi Zhao, Jia Wang, Wei Zheng, Yuanyuan Kong, Guanghui Li

**Affiliations:** 1 Division of Endocrinology and Metabolism, Department of Obstetrics, Beijing Obstetrics and Gynecology Hospital, Capital Medical University, Beijing Maternal and Child Health Care Hospital, Beijing, China; 2 Department of Newborn Screening Center, Beijing Obstetrics and Gynecology Hospital, Capital Medical University, Beijing Maternal and Child Health Care Hospital, Beijing, China

**Keywords:** Thyroid cancer, Pregnancy outcome, Neonatal thyroid function

## Abstract

**Background:**

Evidence regarding adverse pregnancy outcomes in patients with thyroid cancer has been conflicting, and the effect of thyroid dysfunction caused by thyroid hormone suppression therapy in terms of neonatal thyroid stimulating hormone (TSH) is unclear. This study aimed to investigate whether thyroid cancer was associated with adverse pregnancy outcomes and had an adverse effect on neonatal thyroid function.

**Methods:**

This was a retrospective study of 212 singleton pregnancies with thyroid cancer and 35,641 controls without thyroid cancer. Data on maternal pregnancy outcomes and neonatal outcomes were analyzed.

**Results:**

The median TSH level in the thyroid cancer group was significantly lower than that in the control group (0.87 µIU/mL vs. 1.17 µIU/mL; *P* < 0.001), while the FT4 level was higher than that in the control group (17.16 pmol/L vs. 16.33 pmol/L; *P* < 0.001). The percentage of thyroid peroxidase antibodies (TPOAb) positive in the thyroid cancer group was significantly higher than that in the control group (25.0% vs. 11.8%; *P* < 0.001). Pregnancies with thyroid cancer had a higher risk of late miscarriage (OR 7.166, 95% CI: 1.521, 33.775, *P* = 0.013), after adjusting maternal TPOAb positive, there was no statistical significance (OR 3.480, 95% CI: 0.423, 28.614, *P* = 0.246). Pregnancies with thyroid cancer had higher gestational weight gain (GWG) (14.0 kg vs. 13.0 kg, *P* < 0.001). Although there was no significant difference in the prevalence of gestational diabetes mellitus (GDM) (20.8% vs. 17.4%, *P =* 0.194), the oral glucose tolerance test (OGTT) showed that fasting plasma glucose and 2-hour value in the thyroid cancer group were higher than those in the control group (*P* = 0.020 and 0.004, respectively). There was no statistically significant difference in TSH between the thyroid cancer group and the control group, regardless of full-term newborns or preterm newborns.

**Conclusions:**

Thyroid cancer might not have substantial adverse effects on pregnancy outcomes except for excessive GWG. No adverse effect on neonatal TSH was found, but the effect on long-term thyroid function and neuropsychological function in offspring need further study.

**Trial registration:**

Beijing Birth Cohort Study (ChiCTR220058395).

**Supplementary Information:**

The online version contains supplementary material available at 10.1186/s12884-023-05588-4.

## Background

Thyroid cancer is one of the most common cancers diagnosed in young women [1]. Figures from the National Cancer Center (NCC) of China annually report show that thyroid cancer experienced the largest increase in incidence among all cancer types in China and is the fourth most common cancer in Chinese women with age-standardized incidence rates of thyroid cancer at 17.7% [2]. Differentiated thyroid cancer (DTC) is the most frequent subtype of thyroid cancer. In most patients, the standard treatment (thyroidectomy with or without radioactive iodine treatment) is effective [3, 4]. Thyroid hormone replacement therapy or thyroid hormone suppression therapy after thyroidectomy is needed [3, 4]. The main challenge in caring for women with previously treated thyroid cancer is maintaining the thyroid stimulating hormone (TSH) level within the suitable range. Thyroid hormones play an essential role in maintaining pregnancy and promoting optimal fetal development. According to the American Thyroid Association (ATA) recommendation, pregnant women with thyroid cancer should be managed at the same TSH goal as determined preconception [5]. The importance of adequate thyroid hormonal status during pregnancy has been emphasized in association with pregnancy outcomes [6–10]. Several retrospective studies have discussed the effects of a history of thyroid cancer on pregnancy outcomes [11–15]. However, the evidence regarding adverse pregnancy outcomes in patients with thyroid cancer has been conflicting.

The formation and maturation of the newborn’s hypothalamic-pituitary-thyroid axis begin in utero with fetal dependence on maternal thyroid hormones early in the pregnancy and later on the direct supply of iodine from the mother for its thyroxine production [16]. Studies have suggested that maternal thyroid dysfunction during development may induce a shift in the hypothalamic-pituitary-thyroid axis set point of the offspring [17–19]. It has been shown that maternal thyroid function was positively associated with offspring thyroid function at birth [19–22]. Nevertheless, the effect of thyroid dysfunction caused by thyroid hormone suppression therapy in terms of neonatal TSH is unclear.

Therefore, we aimed to evaluate the incidence of adverse pregnancy outcomes, including late miscarriage, stillbirth, and preterm delivery, in patients with thyroid cancer and to investigate whether patients with thyroid cancer had an increased risk of adverse pregnancy outcomes compared with those without thyroid cancer by conducting a retrospective study. Another aim of this study was to investigate whether the neonatal thyroid function of mothers with thyroid cancer differed from that of mothers without thyroid cancer.

## Methods

### Study design

The study was embedded in the Beijing Birth Cohort Study (ChiCTR220058395). The study included singleton pregnancy women who had a history of thyroid cancer or were diagnosed with thyroid cancer during pregnancy and received prenatal care at Beijing Obstetrics and Gynecology Hospital, Capital Medical University from January 2016 to December 2021. The control group was singleton pregnancy women who received care at Beijing Obstetrics and Gynecology Hospital between January 2016 to December 2021, with no history of thyroid cancer, other thyroid diseases, or other malignant tumors. Supplementary Fig. 1 shows the flow chart of the control group’s enrollment. This study has been performed in accordance with the Declaration of Helsinki and has been approved by the ethics committee of Beijing Obstetrics and Gynecology Hospital, Capital Medical University (2018-ky-009-01). Since the anonymous data were respectively extracted from the electronic record without any information that could identify particular individual, a waiver of informed consent was granted by the ethics committee of Beijing Obstetrics and Gynecology Hospital, Capital Medical University.

### Data collection

Baseline information, pregnancy outcomes, and neonatal data were obtained from the electronic database of Beijing Obstetrics and Gynecology Hospital, Capital Medical University. History of abnormal pregnancy includes early and late miscarriage, fetal loss, stillbirth, and preterm delivery. Thyroid cancer-related data were collected, including diagnosis time, duration, treatment modality, and levothyroxine (L-T4) treatment before and during pregnancy. The levels of maternal TSH, free thyroxine (FT4), and thyroid peroxidase antibodies (TPOAb) were recorded during the first prenatal visit before 16 weeks of gestation. The TPOAb concentration was considered positive when levels were above 60 U/mL. Plasma glucose and lipids were measured in the first trimester (before 16 weeks) and the third trimester (32–34 weeks). The 2-h 75-g oral glucose tolerance test (OGTT) was performed at 24–28 weeks’ gestation. In their offspring, TSH was measured in plantar blood samples on filter paper cards screening for congenital hypothyroidism 72 h to 7 days after birth.

### Outcome measures

Maternal outcomes of this study included late miscarriage (defined as the spontaneous loss of a pregnancy from 12 to 23^+ 6^ weeks of gestation) [23], stillbirth (defined as a baby who dies after 28 weeks of pregnancy, but before or during birth), preterm delivery (between gestational weeks 28 and 36^+ 6^); delivery by cesarean section; gestational diabetes mellitus (GDM, diagnosed according to a the 2-h 75-g OGTT between 24 and 28 week’s gestation, with plasma glucose thresholds for fasting, 1-, and 2- hour being 5.1, 10.0, and 8.5 mmol/L, respectively); hypertensive disorders in pregnancy (HDP, including chronic hypertension, gestational hypertension, preeclampsia/eclampsia, and preeclampsia superimposed on chronic hypertension) [24]; premature rupture of membranes (defined as rupture of membranes before the onset of labor) [25]; placenta previa (defined as the placenta lies directly over the internal os) [26]; placenta abruption (defined as the early separation of a placenta from the lining of the uterus before completion of the second stage of labor) [27]; abnormally invasive placentation (defined as previously described by Collins et al. [28]); postpartum hemorrhage (≥ 1000 mL for cesarean delivery or ≥ 500 mL for vaginal delivery). The neonatal outcomes of this study included macrosomia (defined as a newborn birth weight of more than 4000 g); low birth weight infant (LBW, defined as a newborn birth weight below 2500 g).

### Statistical analyses

Quantitative data with non-normal distribution are shown as median (interquartile range), and categorical data are presented as percentages. Mann-Whitney U test, chi-square test, and general linear repeated-measures model were used to assess the differences between the control and study groups where appropriate. Multivariable logistic regression analysis was used to estimate the odds ratio (OR) and the confidence intervals (CIs) for the association of thyroid cancer with adverse pregnancy outcomes. For multivariable logistic analyses, a fixed set of known risk factors for adverse pregnancy outcomes was adjusted for potential confounding, including maternal TPOAb positive, maternal age, multiparity, pre-pregnancy diabetes mellitus (DM), and HDP. Multiple linear regression models were used to investigate the association between variables and GWG. Covariates included the maternal history of thyroid cancer, maternal age, maternal pre-pregnancy body mass index (BMI), multiparity, history of the polycystic ovarian syndrome (PCOS), pre-pregnancy DM, GDM, HDP, and gestational age of delivery. TSH and FT4 were log-transformed and were approximately normally distributed with these transformations. Multiple linear regression models were used to investigate the association between variables and neonatal TSH. Covariates were selected based on biological confounding plausibility, change in effect estimate of the variable of interest, or the reduction of the residual variance of the model. Covariates included the maternal history of thyroid cancer, maternal TSH, maternal FT4, maternal TPOAb positive, maternal age, maternal pre-pregnancy BMI, mode of conception, mode of delivery, birth month, and neonatal gender. When comparing neonatal TSH levels, we used propensity score matching to eliminate possible confounding factors. A 1:1 matching was performed on the propensity score with a maximum caliber of 0.05, and 205 cases in the thyroid cancer group and 205 controls were included in the statistical analysis. Matching variables include maternal age, pre-pregnancy BMI, gestational age of delivery, mode of delivery, birth month, neonatal gender, maternal history of pre-pregnancy DM, GDM, HDP. A *P* value < 0.05 was considered statistically significant. All analyses were performed using Statistical Package of Social Sciences version 25.0 for Windows (SPSS Inc).

## Results

### Population characteristics

From 2016 to 2021, 212 women with thyroid cancer were included in this study. Table 1 shows information about the thyroid cancer therapy of the patients. The median interval from diagnosis of thyroid cancer to conception was 3.0 (1.5, 4.0) years. Only two women were diagnosed during pregnancy. Most patients (98.1%) received thyroidectomy, among whom seven patients (3.3%) were treated with radioactive iodine therapy (RAIT) after surgery. Four patients were on an active surveillance management approach because of their papillary microcarcinomas without clinically evident metastases or local invasion. The median dosage of L-T4 pre-pregnancy was 1.63 µg/kg/day and it increased to 1.77 µg/kg/day during pregnancy. The median TSH and FT4 levels during early pregnancy were 0.87 µIU/mL and 17.16 pmol/L, respectively.

**Table 1:** Information about thyroid cancer therapy (n = 212)

Variables	n (%)/median (IQR)
Time interval between year of diagnosis and conception, years	3.0 (1.5, 4.0)
Cases diagnosed during pregnancy, n (% of pregnancies)	2 (0.9%)
Treatment	
Thyroidectomy only, n (% of pregnancies) Thyroidectomy and RAIT, n (% of pregnancies) No surgery or RAIT, n (% of pregnancies)	201 (94.8%) 7 (3.3%) 4 (1.9%)
Dosage of L-T4 pre-pregnancy, µg/kg/day	1.63 (1.29, 1.94)
Dosage of L-T4 during pregnancy, µg/kg/day	1.77 (1.38, 2.38)

Abbreviations: IQR, inter-quartile range; RAIT, radioactive iodine therapy; L-T4, levothyroxine

The basic characteristics of the two groups are presented in Table 2. Women with thyroid cancer tended to be older with a higher pre-pregnancy BMI and a higher prevalence of pre-pregnancy hypertension. The proportion of women with history of abnormal pregnancy in the thyroid cancer group was significantly higher than that in the control group (19.3% vs. 11.7%; *P =* 0.001). In terms of pre-pregnancy DM, history of HDP, history of GDM, and conceived by in vitro fertilization-embryo transfer (IVF-ET), no difference was observed between thyroid cancer patients and the control group.

**Table 2:** Baseline clinical characteristics of pregnancies

Variables	Thyroid cancer (n = 212)	Control group (n = 35,641)	*P* value
Age, years	32.8 (30.2, 35.4)	31.4 (28.9, 34.5)	< 0.001
Multiparity, n (% of pregnancies)	62 (29.2%)	11,252 (31.6%)	0.466
History of abnormal pregnancy ^a^, n (% of pregnancies)	41 (19.3%)	4165 (11.7%)	0.001
Pre-pregnancy BMI, kg/m^2^	22.0 (20.1, 24.4)	21.2 (19.5, 23.4)	< 0.001
Pre-pregnancy hypertension, n (% of pregnancies)	5 (2.4%)	222 (0.6%)	0.012
Pre-pregnancy DM, n (% of pregnancies)	1 (0.5%)	162 (0.5%)	0.382
History of PCOS, n (% of pregnancies)	5 (2.4%)	1449 (4.1%)	0.209
History of HDP, n (% of multiparity)	3 (4.8%)	437 (3.9%)	0.735
History of GDM, n (% of multiparity)	6 (9.7%)	883 (7.8%)	0.632
Family history of hypertension, n (% of pregnancies)	55 (25.9%)	6536 (18.3%)	0.004
Family history of DM, n (% of pregnancies)	32 (15.1%)	4092 (11.5%)	0.100
Conceived by IVF-ET, n (% of pregnancies)	11 (5.2%)	1635 (4.6%)	0.677
Thyroid function during early pregnancy			
TSH, µIU/mL FT4, pmol/L TPOAb positive, n (% of pregnancies)	0.87 (0.30, 1.70) 17.16 (15.62, 19.11) 53 (25.0%)	1.20 (0.67, 1.87) 15.48 (14.22, 16.93) 4190 (11.8%)	< 0.001 < 0.001 < 0.001

Abbreviations: BMI, body mass index; DM, diabetes mellitus; PCOS, polycystic ovary syndrome; HDP, hypertensive disorders of pregnancy; GDM, gestational diabetes mellitus; IVF-ET, in vitro fertilization-embryo transfer

^a^ History of abnormal pregnancy included early and late miscarriage, fetal loss, stillbirth, and preterm delivery

When comparing the differences in the maternal thyroid function during early pregnancy between the two groups, we found that the median TSH level in the thyroid cancer group was significantly lower than that in the control group (0.87 µIU/mL vs. 1.17 µIU/mL; *P* < 0.001), while the FT4 level in the thyroid cancer group was significantly higher than that in the control group (17.16 pmol/L vs. 16.33 pmol/L; *P* < 0.001). The percentage of TPOAb positive in pregnant women with thyroid cancer was 25.0%, which was significantly higher than that in the control group (11.8%), *P* < 0.001.

### Maternal outcomes

The maternal outcomes between pregnant women with and without thyroid cancer was shown in Table 3. Most adverse obstetric outcomes such as stillbirth, preterm delivery, cesarean section, GDM, HDP, premature rupture of membranes, placenta previa, placenta abruption, abnormally invasive placentation, and postpartum hemorrhage had no difference between the two groups. Pregnant women with thyroid cancer had a higher risk of late miscarriage (0.9% vs. 0.0%; OR 7.166, 95% CI: 1.521, 33.775, *P* = 0.013). However, after adjusting maternal TPOAb positive, there was no statistical significance (OR 3.480, 95% CI: 0.423, 28.614, *P* = 0.246) (Table 4).

**Table 3:** The maternal and neonatal outcomes between pregnancies with and without thyroid cancer

Variables	Thyroid cancer (n = 212)	Control group (n = 35,641)	*P* value
Gestational weeks of deliveries, weeks	39.0 (38.0, 40.0)	39.4 (38.6, 40.3)	0.816
GWG of all pregnancies, kg	14.0 (10.9, 16.0)	13.0 (9.0, 16.0)	0.012/ <0.001^a^
GWG of full-term deliveries, kg	14.0 (11.0, 16.3)	13.0 (9.5, 16.0)	0.011/ <0.001^a^
Late miscarriage, n (% of pregnancies)	2 (0.9%)	4 (0.0%)	0.001
Stillbirth, n (% of pregnancies)	2 (0.9%)	62 (0.2%)	0.055
Preterm deliveries, n (% of pregnancies)	9 (4.2%)	1706 (4.8%)	0.713
Cesarean section, n (% of deliveries)	83 (39.9%)	12,182 (34.2%)	0.086
GDM, n (% of pregnancies)	44 (20.8%)	6188 (17.4%)	0.194
HDP, n (% of pregnancies)	21 (9.9%)	2583 (7.2%)	0.137
Premature rupture of membranes, n (% of pregnancies)	54(25.5%)	10,927 (30.7%)	0.102
Placenta previa, n (% of pregnancies)	3(1.4%)	349 (1.0%)	0.468
Placenta abruption, n (% of pregnancies)	5(2.4%)	522 (1.5%)	0.246
Abnormally invasive placentation, n (% of pregnancies)	4(1.9%)	1006 (2.8%)	0.412
Postpartum hemorrhage, n (% of pregnancies)	46(21.7%)	6918 (19.4%)	0.401
Boy, n (% of deliveries)	105 (49.5%)	18,309 (51.4%)	0.593
Birthweight, g	3365 (3051, 3680)	3370 (3090, 3650)	0.823
Macrosomia, n (% of deliveries)	15 (7.1%)	2542 (7.1%)	0.974
LBW, n (% of deliveries)	9 (4.2%)	1143 (3.2%)	0.393
Neonatal TSH, µIU/mL	1.93 (1.31, 2.91)	1.79 (1.24, 2.67)	0.145
Full-term neonatal TSH, µIU/mL	1.93 (1.31, 2.92)	1.79 (1.24, 2.67)	0.121
Preterm neonatal TSH, µIU/mL	1.53 (1.25, 2.64)	1.86 (1.34, 2.62)	0.726

Abbreviations: GWG, gestational weight gain; GDM, gestational diabetes mellitus; HDP, hypertensive disorders of pregnancy; LBW, low birth weight

^a^ Adjusted for maternal age, pre-pregnancy BMI, and gestational week of delivery

**Table 4:** Multivariable logistic regression results for the associations between thyroid cancer and adverse pregnancy outcomes

Dependent variable	ORs (95% CI)	*P* value
Late miscarriage	3.480 (0.423, 28.614)	0.246 ^a^
Stillbirth	2.230 (0.528, 9.413)	0.275 ^a^
Preterm delivery	0.509 (0.207, 1.254)	0.142 ^a^
GDM	1.187 (0.786, 1.792)	0.416 ^b^
HDP	1.396 (0.828, 2.351)	0.210 ^b^
Premature rupture of membranes	0.713 (0.501, 1.016)	0.061 ^a^
Placenta previa	1.277 (0.307, 5.304)	0.736 ^a^
Placenta abruption	1.778 (0.715, 4.422)	0.215 ^a^
Abnormally invasive placentation	0.230 (0.032, 1.656)	0.230 ^a^
Postpartum hemorrhage	0.932 (0.641, 1.356)	0.712 ^a^
Macrosomia	1.262 (0.729, 2.183)	0.406 ^c^
LBW	0.895 (0.415, 1.933)	0.778 ^c^

Abbreviations: GDM, gestational diabetes mellitus; HDP, hypertensive disorders of pregnancy; LBW, low birth weight

^a^ Adjusted for maternal TPOAb positive, maternal age, multiparity, pre-pregnancy DM, GDM, and HDP.

^b^ Adjusted for maternal TPOAb positive, maternal age, multiparity, pre-pregnancy BMI.

^c^ Adjusted for maternal TPOAb positive, maternal age, multiparity, pre-pregnancy BMI, neonatal sex

### The differences in GWG and glucolipid levels

As shown in Table 3, no matter whether in all pregnant women or full-term pregnant women, the GWG in the thyroid cancer group was higher than that in the control group. Multiple linear regression analysis showed that GWG was related to the history of thyroid cancer after adjusting maternal age, pre-pregnancy BMI, multiparity, history of PCOS, pre-pregnancy DM, GDM, HDP, and delivery gestational age (Table 5).

**Table 5:** Multiple linear regression results for the association between GWG and related factors

Dependent variable	Unstandardized coefficients		Standardized coefficients	*P* value
	B	Std. Error	Beta	
History of thyroid cancer	1.336	0.348	0.044	< 0.001
Age at conception	-0.064	0.016	-0.049	< 0.001
Pre-pregnancy BMI	-0.334	0.019	-0.210	< 0.001
Multiparity	-0.507	0.122	-0.051	< 0.001
History of PCOS	0.119	0.250	0.005	0.635
Pre-pregnancy DM	2.184	0.812	0.031	0.007
Gestational diabetes	2.231	0.160	0.163	< 0.001
HDP	-0.753	0.196	-0.045	< 0.001
Delivery gestational age	0.176	0.055	0.037	0.001

Abbreviations: GWG, gestational weight gain; BMI, body mass index; PCOS, polycystic ovary syndrome; DM, diabetes mellitus; HDP, hypertensive disorders of pregnancy

When comparing the differences in the glucolipid levels in the first and third trimesters of pregnancy between the two groups (Supplementary Table 1), levels of total cholesterol (TCHO) and high-density lipoprotein cholesterol (HDL-C) in the thyroid cancer group were found to be significantly higher in the first trimester. However, there was no significant difference in glucolipid levels between the two groups in the third trimester. Although there was no significant difference in the prevalence of GDM between the two groups (20.8% vs. 17.4%; *P* = 0.194), OGTT showed that fasting plasma glucose and 2-hour value in the thyroid cancer group were higher than those in the control group (*P* = 0.020 and = 0.004, respectively) (Supplementary Table 1).

### Neonatal outcomes

As shown in Table 3, the proportions of male newborns in the thyroid cancer group and the control group were 49.5% and 51.4%, respectively. The mean newborn birthweight of the thyroid cancer group was 3365 g which was similar to that of the control group (3370 g). And there was no significant difference in the proportion of macrosomia and the proportion of LBW between the two groups.

### The differences in neonatal TSH levels

We found that there was no statistically significant difference in TSH of full-term newborns and preterm newborns between the thyroid cancer group and the control group (Table 3). We got the same results after propensity score matching (1:1) (Supplementary Table 2). Matching variables include maternal age, pre-pregnancy BMI, gestational age of delivery, mode of delivery, birth month, neonatal gender, maternal history of pre-pregnancy DM, GDM, and HDP. Multiple linear regression analysis showed that neonatal TSH was associated with maternal TSH, maternal FT4, maternal age, pre-pregnancy BMI, mode of delivery, and birth month (Table 6). No correlation was found between neonatal TSH and maternal history of thyroid cancer (Table 6).

**Table 6:** Multiple linear regression results for the association between neonatal TSH and related factors

Dependent variable	All deliveries				Full-term deliveries			
	Unstandardized coefficients		Standardized coefficients	*P* value	Unstandardized coefficients		Standardized coefficients	*P* value
	B	Std. Error	Beta		B	Std. Error	Beta	
Maternal history of thyroid cancer	-0.026	0.020	-0.015	0.184	-0.027	0.020	-0.015	0.185
Maternal TSH	0.048	0.007	0.084	< 0.001	0.047	0.008	0.082	< 0.001
Maternal FT4	0.220	0.057	0.051	< 0.001	0.223	0.059	0.051	< 0.001
Maternal TPOAb positive	-0.003	0.009	-0.004	0.712	-0.001	0.009	-0.002	0.891
Maternal age	-0.002	0.001	-0.033	0.004	-0.002	0.001	-0.033	0.005
Maternal pre-pregnancy BMI	-0.002	0.001	-0.023	0.042	-0.002	0.001	-0.024	0.045
Fertilization mode	-0.020	0.013	-0.017	0.135	-0.023	0.014	-0.019	0.105
Delivery mode	0.070	0.004	0.185	< 0.001	0.073	0.004	0.191	< 0.001
Month of birth	0.003	0.001	0.037	0.001	0.003	0.001	0.034	0.004
Neonatal gender	-0.010	0.006	-0.019	0.089	-0.013	0.006	-0.024	0.036

Abbreviations: TSH, thyroid stimulating hormone; FT4, free thyroxine; TPOAb, thyroid peroxidase antibody; BMI, body mass index

## Discussion

Pregnant women with a history of thyroidectomy with or without radioactive iodine treatment were treated with supplementary LT4 throughout pregnancy. In this study, thyroid function status was significantly different in women with thyroid cancer in early pregnancy. However, the disease might not have substantial adverse effects on pregnancy outcomes except for GWG. Women with thyroid cancer had an excessive GWG. Considering that maternal thyroid function is a determinant of newborn thyroid function [20], we compared neonatal TSH levels between pregnancies with thyroid cancer and their controls. However, we found no significant difference in the level of neonatal TSH between the two groups. Further analysis observed an association between neonatal TSH and maternal FT4 and TSH levels in the first trimester of pregnancy, but no correlation was found between neonatal TSH and maternal history of thyroid cancer.

In this study, the proportion of late miscarriages of pregnant women with thyroid cancer was higher than that of their controls (0.9% vs. 0.0%; *P* = 0.001). A meta-analysis also demonstrated an increased risk of miscarriage or abortion in women with thyroid cancer [29]. But the authors considered the result might be biased because of the small number of studies and considering the potentially significant publication bias. The percentage of TPOAb positive in pregnant women with thyroid cancer was significantly higher than that in the control group (25.0% vs. 12.9%; *P* < 0.001). After adjusting maternal TPOAb positive, the odds of late miscarriage with thyroid cancer were 3.480 (0.423, 28.614) and there was no statistical significance. There is evidence that the presence of maternal thyroid autoantibodies is strongly associated with miscarriage [30, 31], and animal study has found an increased proportion of fetal resorption in mice with induced thyroperoxidase antibodies [32]. Considering the limited number of available patients, further studies are warranted to explore whether TPOAb is associated with increased late miscarriage in pregnant women with thyroid cancer.

The thyroid hormone has an important role in regulating metabolism, which influences key metabolic pathways that control energy balance. Our present study showed that pregnant women with thyroid cancer had an increased GWG. Liu et al. did not find differences in GWG between thyroid cancer survivors and the control group [11]. However, a propensity score-matched cohort study showed that thyroid cancer survivors had reduced weight gain during pregnancy [14]. Owing to the evidence regarding GWG in patients with thyroid cancer being few and conflicting, further studies are warranted to strengthen these conclusions. It has been reported that GWG may be related to the risk of pregnancy complications [33, 34]. Moreover, thyroid dysfunction has been suggested to play a role in the etiology of GDM [35–39]. There was no evidence of increased risk of GDM in patients with thyroid cancer confirmed by our present study and other studies [11, 14]. However, OGTT showed that fasting plasma glucose and 2-hour value in the thyroid cancer group were higher than those in the control group. No increased incidence of other major adverse pregnancy outcomes such as macrosomia, preterm delivery, and cesarean delivery was detected among women with thyroid cancer in our study. We analyzed the difference in total GWG rather than that of pre-OGTT weight gain, and the latter has a better correlation with the OGTT results. Whether there is a causal relationship between excessive GWG and the difference in OGTT needs to be further explored. Current evidence suggests that clinicians should pay attention to GWG in women with thyroid cancer.

Regarding other pregnancy outcomes assessed in our study, we found no statistically significant difference between the thyroid cancer group and the control group. The evidence regarding adverse pregnancy outcomes in patients with thyroid cancer has been conflicting. A retrospective cohort study reported by Liu et al. showed that pregnant women with a history of thyroid cancer had a higher risk of abnormally invasive placentation [11]. Another retrospective cohort study revealed that pregnancies complicated by thyroid cancer have higher incidences of venous thromboembolism and need for transfusions, with comparable overall newborn outcomes [40]. A study conducted by Cho and colleagues showed that women with a history of thyroid cancer had a slightly higher risk of preterm birth, high birth weight, preeclampsia, postpartum hemorrhage, and placenta previa [13]. The results of the meta-analysis showed that the event rates for preterm labor and congenital anomalies in patients with DTC who underwent thyroidectomy were 0.07 (95% CIs, 0.05–0.09; 14 studies) and 0.03 (95% CIs, 0.02–0.06; 17 studies), respectively, which were similar to those previously reported in the general population [29]. These valuable data should reassure both patients and physicians that a history of thyroid cancer does not meaningfully impact the risk of adverse pregnancy outcomes.

Previous studies have shown that maternal thyroid function is positively associated with offspring thyroid function at birth [20, 21]. Our present showed that the thyroid function status was significantly different in women with thyroid cancer in early pregnancy, which was consistent with other studies [11, 41]. João Anselmo et al. conducted a retrospective study concerning fetal loss associated with excess thyroid hormone exposure [42]. Individuals with resistance to thyroid hormone (RTH) achieve euthyroidism by maintaining high serum levels of free thyroid hormone. They found that the levels of TSH in unaffected infants born to RTH mothers were suppressed, which indicated that the high maternal thyroid hormone levels produce fetal thyrotoxicosis [42]. Another study showed that the amniotic fluid thyroxine level in patients with thyroid carcinoma who were placed on suppressive thyroxine treatment was lower than that in normal pregnancies and hypothyroid patients [43]. Fan et al. also found that levels of neonatal TSH in the thyroid cancer group were significantly lower than those in the control group [41]. However, we found no significant difference in the levels of neonatal TSH between the thyroid cancer group and the control group. Considering the limited number of available evidence, further studies are warranted to investigate the effect of LT4 suppression therapy during pregnancy on neonatal thyroid function in women with thyroid cancer.

We have for the first time performed a detailed analysis of glucolipid metabolism which deserves attention for pregnancies with thyroid cancer in the future. And our conclusion should reassure both patients and physicians that thyroid cancer does not meaningfully impact the risk of adverse pregnancy outcomes. Our study was limited by the retrospective and single-center study design. With regard to 212 women with thyroid cancer, more information could not be available, including initial risk stratification and response to therapy. Moreover, the laboratory data on thyroid function in the second and third trimesters especially in pregnancies without thyroid cancer were not available. Data on cases of miscarriage before 12 weeks of gestation were not included in the electronic database of our hospital. Therefore, we could not analyze whether pregnant women with thyroid cancer had an increased risk of early miscarriage. The lack of data on the Apgar score and the percentage of admissions to the neonatal unit was another limitation of our study.

## Conclusions

In conclusion, thyroid cancer might not have substantial adverse effects on pregnancy outcomes except for excessive GWG. Although there was no significant difference in the prevalence of GDM, pregnant women with thyroid cancer had higher fasting plasma glucose and a 2-hour value in OGTT. Thyroid function status was significantly different in women with thyroid cancer in early pregnancy, however, we found no significant effect on neonatal TSH. The effect on long-term thyroid function and neuropsychological function in offspring need further study.

## Electronic supplementary material

Below is the link to the electronic supplementary material.

Additional file 1

## Data Availability

The datasets analyzed during the current study are available from the corresponding author on reasonable request.

## Abbreviations

ATA: The American Thyroid Association
BMI: Body mass index
CIs: Confidence intervals
DM: Diabetes mellitus
DTC: Differentiated thyroid cancer
FT4: Free thyroxine
GDM: Gestational diabetes mellitus
GWG: Gestational weight gain
HDL-C: High-density lipoprotein cholesterol
HDP: Hypertensive disorders in pregnancy
IVF-ET: In vitro fertilization-embryo transfer
LBW: Low birth weight
L-T4: Levothyroxine
NCC: The National Cancer Center
OGTT: Oral glucose tolerance test
OR: Odds ratio
PCOS: Polycystic ovarian syndrome
RAIT: Radioactive iodine therapy
RTH: Resistance to thyroid hormone
TCHO: Total cholesterol
TPOAb: Thyroid peroxidase antibodies
TSH: Thyroid stimulating hormone
