# Supplementary material for: Pregnancy outcomes and neonatal thyroid function in women with thyroid cancer: a retrospective study
Source: BMC Pregnancy Childbirth. 2023 May 25;23:383. doi: 10.1186/s12884-023-05588-4 (PMC10210488; doi:10.1186/s12884-023-05588-4)
Supplement: Supplementary file 1 — Additional file 1 [file 12884_2023_5588_MOESM1_ESM.docx]

**Supplementary Figures**

**
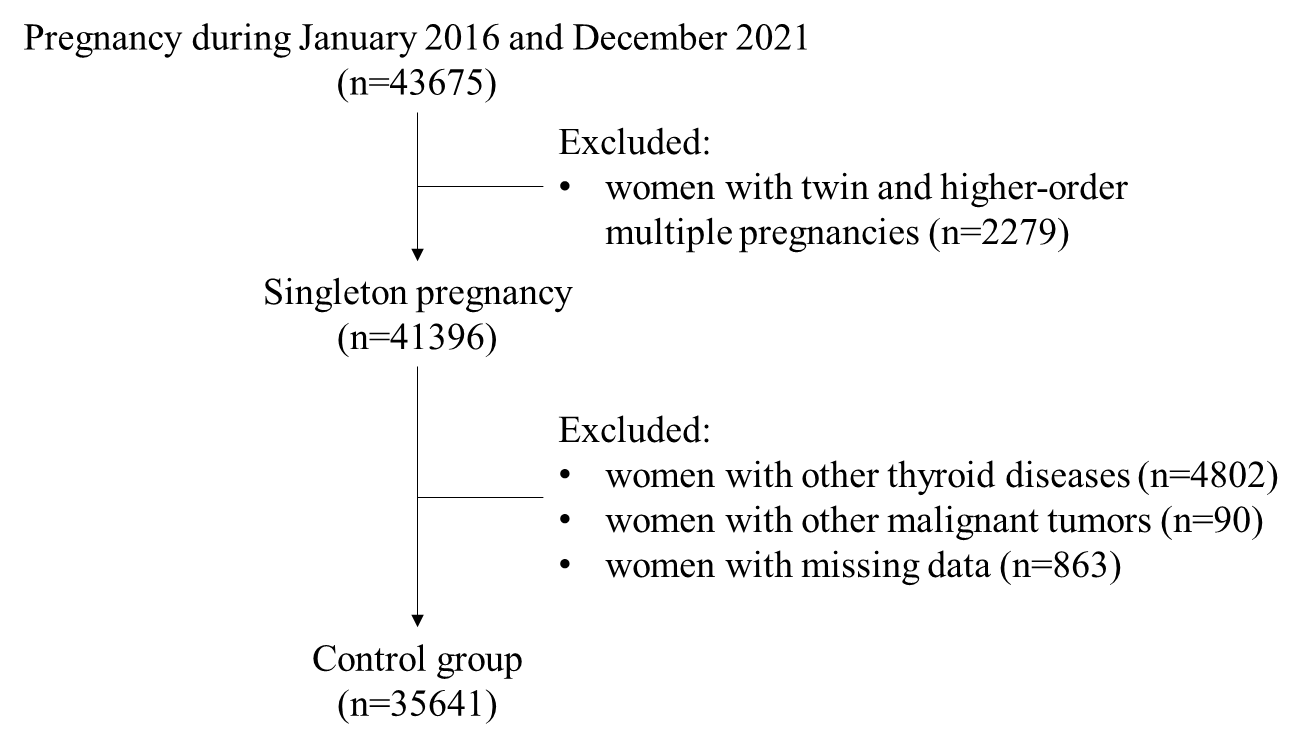
**

Supplementary Figure 1. Flow chart of the enrollment of the control group.

**Supplementary Tables**

Supplementary Table 1. The differences in the glucolipid levels between pregnancies with and without thyroid cancer.

| Variables | | Thyroid cancer  (n=212) | Control group (n=35641) | *P* value |
| --- | --- | --- | --- | --- |
| First trimester | GLU, mmol/L | 4.68 (4.45, 4.94) | 4.63 (4.41, 4.86) | 0.099/ 0.507^a^ |
|  | TCHO, mmol/L | 4.39 (3.83, 4.91) | 4.17 (3.76, 4.63) | 0.001/ 0.004^a^ |
|  | TG, mmol/L | 1.04 (0.81, 1.40) | 0.97 (0.76, 1.27) | 0.008/ 0.204^a^ |
|  | HDL-C, mmol/L | 1.53 (1.32, 1.77) | 1.50 (1.31, 1.70) | 0.189/ 0.014^a^ |
|  | LDL-C, mmol/L | 2.34 (1.92, 2.72) | 2.17 (1.82, 2.56) | 0.002/ 0.053^a^ |
| OGTT | GLU (0h), mmol/L | 4.48 (4.25, 4.75) | 4.46 (4.24, 4.71) | 0.020 |
|  | GLU (1h), mmol/L | 7.60 (6.70, 8.94) | 7.60 (6.45, 8.81) | 0.284 |
|  | GLU (2h), mmol/L | 6.77 (5.94, 7.85) | 6.47 (5.66, 7.43) | 0.004 |
| Last trimester | FBG, mmol/L | 4.31 (4.07, 4.67) | 4.31 (4.06, 4.59) | 0.286 |
|  | TCHO, mmol/L | 6.50 (5.71, 7.17) | 6.34 (5.63, 7.08) | 0.365 |
|  | TG, mmol/L | 3.06 (2.47, 3.68) | 2.85 (2.32, 3.54) | 0.095 |
|  | HDL-C, mmol/L | 1.78 (1.55, 2.04) | 1.79 (1.57, 2.04) | 0.674 |
|  | LDL-C, mmol/L | 3.48 (2.76, 4.00) | 3.43 (2.82, 4.11) | 0.248 |

Abbreviations: GLU, glucose; TCHO, total cholesterol; TG, triglyceride; HDL-C, high-density lipoprotein cholesterol; LDL-C, low-density lipoprotein cholesterol; OGTT, oral glucose tolerance test.

^a^ Adjusted for maternal age and pre-pregnancy BMI.

Supplementary Table 2. Neonatal TSH between patients with and without thyroid cancer after propensity score matching (1:1).

| Varibales | Thyroid cancer  (n=205) | Control group  (n=205) | *P* value |
| --- | --- | --- | --- |
| Neonatal TSH, μIU/mL | 1.92 (1.32, 2.87) | 2.10 (1.43, 3.21) | 0.105 |
| Full-term neonatal TSH, μIU/mL | 1.93 (1.32, 2.88) | 2.13 (1.37, 3.26) | 0.122 |
| Preterm neonatal TSH, μIU/mL | 1.53 (1.25, 2.64) | 1.88 (1.57, 2.61) | 0.312 |

Abbreviations: TSH, thyroid stimulating hormone.

Matching variables include maternal age, pre-pregnancy BMI, gestational age of delivery, mode of delivery, birth month, neonatal gender, maternal history of pre-pregnancy DM, GDM, HDP.
